# Multiple Recognition-Based Sensor for Pesticide Residues

**DOI:** 10.3389/fchem.2022.856698

**Published:** 2022-03-21

**Authors:** Jie Li, Keren Chen, Longjiao Zhu, Xiangyang Li, Changmo Li, Qiaoying Chang, Wentao Xu

**Affiliations:** ^1^ State Key Laboratory of Food Nutrition and Safety, Tianjin University of Science & Technology, Tianjin, China; ^2^ Key Laboratory of Precision Nutrition and Food Quality, Department of Nutrition and Health, China Agricultural University, Beijing, China; ^3^ Beijing Laboratory for Food Quality and Safety, Food Science and Engineering College, Beijing University of Agriculture, Beijing, China

**Keywords:** pesticide detection, protein, aptamer, nanomaterial, macrocycle

## Abstract

The use of pesticides is gradually increasing to improve the yield and quality of crops. However, excessive pesticide use has led to a dramatic pollution increase in the environment and agricultural products, posing severe human health risks. Therefore, rapid, sensitive pesticide detection is essential. Various pesticides detection methods and products have been developed in recent years. This brief review summarized the point-of-care testing (POCT) detection of pesticides based on multiple recognition, including protein-, aptamer-, nanomaterial-, and macrocycle-based recognition. The review aimed to address the growing demands for regulating and destroying pesticides or other adverse agriculture-related applications in the real world.

## Graphical Abstract

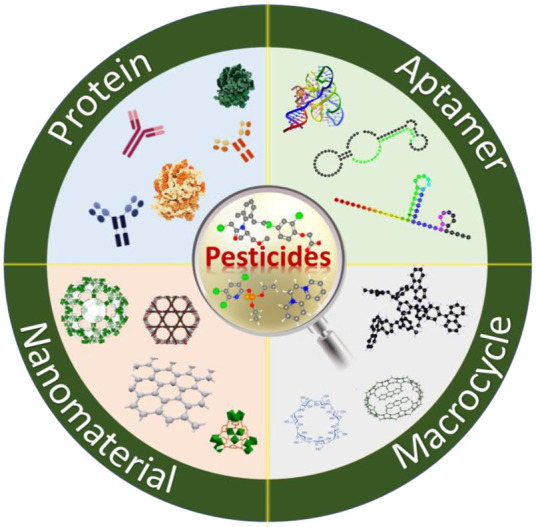

## Introduction

Synthetic organophosphorus pesticides (OPs) emerged in the 1940s and have been widely used since then due to their ideal insecticidal effects (Fenner et al., 2013). The World Health Organization defines pesticides as compounds used in agricultural production to kill insects, fungi, and weeds that damage crops. More than 1,000 kinds of pesticides are currently used worldwide and commonly include insecticides, herbicides, fungicides, and growth regulators. Millions of tons of pesticides are used annually during agricultural production throughout the world. Although pesticides can improve the yield and quality of agricultural products, excessive use harms the environment and organisms. Pesticides can remain in soil and water and enriched in organisms through the food chain. The consumption of food containing excessive pesticides causes symptoms such as vertigo, diarrhea, blindness, or even death (Fang et al., 2020). Therefore, rapid, sensitive, highly specific are necessary for pesticide detection when their use is unavoidable.

Over the past few decades, pesticide detection relied heavily on the utilization of high-performance liquid chromatography (HPLC), mass spectrometry (MS), and gas chromatography (GC). Although these methods are precise, they require expensive instruments, complex samples, and experienced operators. This prompted the development of increasingly rapid pesticide detection techniques. This review introduces pesticide detection applications based on different recognition motifs. The identification strategies can generally be divided into protein-, aptamer-, nanomaterial-, and macrocycle-based recognition. The comparison between different recognition motifs in the detection of pesticide residues is concluded in Table 1.

**TABLE 1:** A comparison between different recognition motifs in the detection of pesticide residues.

Recognition motif	Recognition mechanism	Primary output signal of the biosensor	Advantage	Disadvantage
Protein	Immunological recognition	Colorimetric	Easy modification	Enzyme denaturation
	Enzymatic reaction	Electrochemical	High specificity	Instability
	—	—	—	High cost
Aptamer	Specific structure-based molecular recognition	Colorimetric	Low cost	Nuclease degradation
		Electrochemical	Easy modification	
		Fluorescent	High specificity	
		Chemiluminescent	High affinity	
			Easy preparation	
Nanomaterial	Host-guest interaction	Fluorescent	High stability	Cross-reactivity
		Electrochemical	Easy modification	Low specificity
		—	High reproducibility	—
		—	Easy preparation	—
Macrocycle	Host-guest interaction	Fluorescent	High stability	Low specificity
				Complex preparation

## Protein-Based Recognition of Pesticide Compounds

Protein-based recognition pesticide detection is a commonly used method that involves the interaction between pesticides and enzymes or antibodies (Liu et al., 2017; Fang et al., 2020).

**FIGURE 1:**
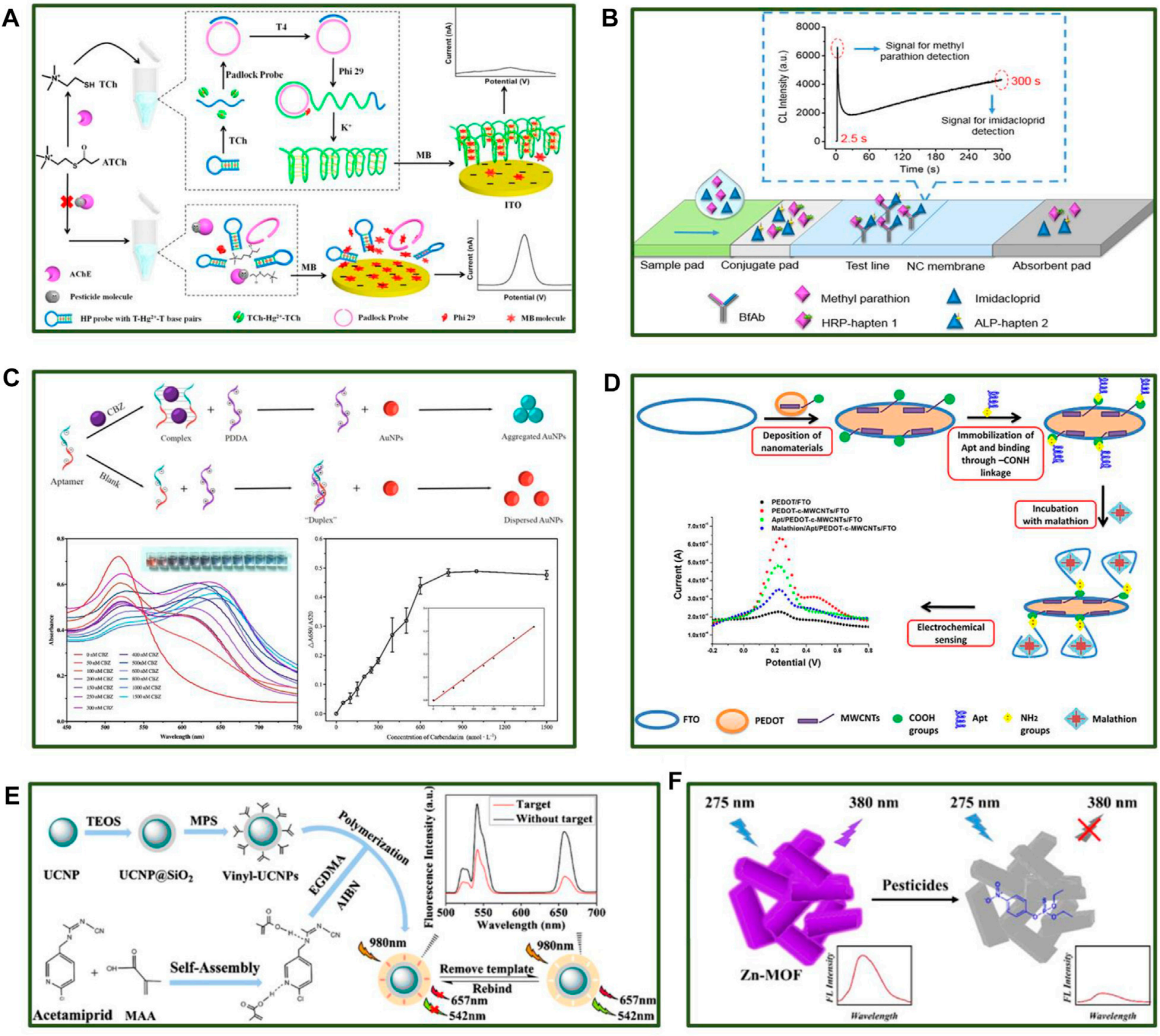
Different applications for the rapid and sensitive detection of pesticide residues based on multiple recognition. **(A)** Enzyme-based recognition biosensors for OPs and carbamate pesticides. **(B)** Test paper for detecting pesticide compounds based on antibodies. **(C)** A schematic of carbendazim (CBZ) detection using a colorimetric sensor (top), the solution absorbance in different CBZ concentrations (bottom left), the detection performance of the colorimetric sensor (bottom right). **(D)** A schematic of malathion detection using an electrochemical sensor. **(E)** The construction of molecularly imprinted upconversion nanoparticles and the detection process. **(F)** The application of Zn-MOF for parathion sensing.

Acetylcholinesterase (AChE) is an important enzyme in biological nerve conduction that can affect the physiological functions of the nervous system when destroyed (Lionetto et al., 2013). Therefore, OPs or carbamate pesticides that target AChE achieve an insecticidal effect by inhibiting AChE activity in insects after ingestion. AChE-based pesticide detection can be realized using pH, fluorescence intensity, absorbance, and electrochemical input signals. AChE-catalytic hydrolysates can induce conformational DNA changes to trigger roll-circle amplification (RCA). A homogeneous electroanalysis platform for pesticide detection was reported in 2017 (Liu et al., 2017). As shown in Figure 1A, the G-quadruplex produced by RCA captured free methylene blue (MB) in the solution without pesticides. Therefore, the electrochemical signal decreased due to reduced MB diffusion to the electrode surface. Since the RCA reaction cannot occur in the presence of pesticides due to AChE destruction, a large quantity of MB makes contact with the electrodes, increasing the electrochemical signal. The pesticide content in a sample can be detected depending on the level of the electrochemical signal. In addition, alkaline phosphatase (ALP), OP hydrolase, tyrosinase enzymes, and laccases can also be used for pesticide detection. Researchers developed a multi-enzyme targeted fluorescent probe to improve pesticide detection efficiency (Guo et al., 2021).

Utilizing immunosensors for pesticide detection is increasing due to their high affinity and specificity. The recognition of pesticides by antibodies can be converted into directly readable signals using a smart mechanism. Immunosensors involving fluorescence, colorimetry, and chemiluminescence are currently used for pesticide detection and divided into sandwich and competitive formats. Due to the specific characteristics of pesticides, they are typically detected using the competitive format. The pesticide molecules in samples compete with enzyme-labeled haptens to bind with antibodies. Therefore, fewer enzyme-labeled pesticide molecules can bind to the antibodies at a higher pesticide content level, leading to lower signal output. Shu et al. (2017) prepared and used a bifunctional antibody to develop immunochromatographic test strips that simultaneously detected methyl parathion and imidacloprid. As shown in Figure 1B, the hapten of methyl parathion was labeled with horseradish peroxidase (HRP) to obtain HRP-hapten 1, while the hapten of imidacloprid was labeled with ALP to produce ALP-hapten 2, which could bind to antibodies fixed on the test line. The co-reactants were then injected, triggering two time-resolved chemiluminescence reactions to generate the signal output. The hapten design is crucial for the detection performance in this assay since it is closely related to sensitivity. Wu et al. (2021) proposed a new synthetic method by coating antigens directly conjugated to the carrier protein to develop a highly sensitive OP detection kit, overcoming the complex hapten synthesis process. Due to the low limit of detection (LOD), impedimetric immunosensors that can achieve label-free detection are also of widespread concern. Fusco et al. (2017) developed an impedimetric immunosensor, which achieved a sensitive detection of 2,4-Dichlorophenoxy Acetic Acid over a wide linear range by immobilizing antibodies on the conductive nanocomposites.

In addition to enzymes and antibodies, peptides can also be used for pesticide detection. Wang J. et al. (2020) label tetraphenylethylene (TEP) with peptides to detect OPs. When OPs are present, they can form adducts with peptides, promoting peptide aggregation and inducing the fluorescence emission enhancement of TEP-peptide.

## Aptamer-Based Recognition of Pesticide Compounds

Nucleic acid aptamers are sequences that specifically identify targets with high affinity and specificity and are typically produced via the systematic evolution of ligands through exponential enrichment (SELEX) screening technique. In 1990, Tuerk & Gold and Ellington & Szostak proposed the SELEX screening method and the concept of the nucleic acid aptamer, respectively (Ellington and Szostak, 1990; Tuerk and Gold, 1990). Since then, aptamers have developed rapidly and are applied in different fields for biosensing, biomedicine, bioimaging, and nanomaterial.

Molecular recognition based on nucleic acid aptamers has attracted significant attention for rapid pesticide detection. The strong specificity and affinity of aptamer and target binding facilitate the identification process when detecting pesticides. Aptamers are ideally equipped to recognize pesticides ranging from polymers to small molecules. Since aptamers are easily modified, they can be combined with many types of signal output units for point-of-care testing (POCT) pesticide detection. Aptamers can be used in optical biosensors, such as colorimetric, fluorescent, and chemiluminescent sensors.

Gold nanoparticles (AuNPs) are either dispersed or aggregated depending on the solution conditions, causing a color change to occur (Mirkin et al., 1996). Therefore, AuNPs are often used in colorimetric pesticide aptasensors. Wang S. et al. (2020) developed an aptasensor consisting of AuNPs and cationic polymer poly-diallyldimethylammonium chloride (PDDA) to detect a broad spectrum of the systemic fungicide, CBZ. As shown in Figure 1C, the AuNPs displayed negatively charged properties in the absence of CBZ, forming a stable complex with the cationic PDDA via electrostatic interaction. The AuNPs appeared as a red solution in a dispersed state. When the target was present in the system, the CBZ aptamer bonded to CBZ with priority, failing to form a stable complex with PDDA to protect the AuNPs from aggregation. The aggregated AuNPs turned the solution blue and enabled CBZ detection based on changes in the absorbance of the solution. The LOD for this strategy was 2.2 nmol L^−1^ with a detection range of 2.2–500 nmol L^−1^. Moreover, this approach exhibited ideal sensitivity and specificity, achieving high CBZ recovery in water samples (94.9–104.8%).

Similarly, the construction of a chemiluminescent sensing platform for acetamiprid detection was achieved based on the high affinity between aptamers and targets as well as the correlation between the morphology of AuNPs and their catalytic effect (Qi et al., 2016). In the presence of H_2_O_2_ and luminol, AuNPs can achieve catalytic action and electrochemiluminescence. Therefore, highly sensitive acetamiprid detection can be achieved at a LOD of 62 pmol L^−1^.

Fluorescence signals represent output signals commonly used in the field of sensing. In biosensors, the signal output can be performed by introducing molecules with fluorescent properties. In 2018, Wangoo and Sharma’s team and Lu’s team achieved rapid marathon and isocarbophos (ICP) detection using different adapters (Bala et al., 2018; Li et al., 2018). Using similar strategies, that is, before and after adding the measured target, changes in the aptamer conformation caused differences in the number of free nucleic acid single-strands in the system. The fluorescence value of the system was modified to realize quantitative pesticide detection based on the interaction with nanomaterials (CdTe@CdS quantum dots or multi-walled carbon nanotubes). Comparatively, the detection performance of the malathion biosensor (LOD 4 pmol L^−1^) was superior to the ICP biosensor (LOD 10 nmol L^−1^).

In addition to optical sensors, electrochemical biosensors based on aptamer recognition can also achieve excellent pesticide detection. In 2019, an aptasensor was developed for malathion detection, consisting of polymer poly (3,4-ethylenedioxythiophene) (PEDOT) and carboxylated multi-walled carbon nanotubes (c-MWCNTs) (Kaur et al., 2019). The aptamers were introduced onto the electrode surface via chemical bonds. This electrochemical sensor achieved ultra-sensitive detection in a linear detection range of 0.1 fmol L^−1^ to 1 μmol L^−1^ with an ideal LOD of 0.5 fmol L^−1^ (Figure 1D). Besides, Xu et al. (2020) provided a creative electrochemical aptasensor containing highly porous gold and aptamer for the impedimetric determination of acetamiprid, which displayed a linear response for target in the concentration range of 0.5–300 nmol L^−1^ with a LOD of 0.34 nmol L^−1^.

Additionally, scattered light signal sensors based on aptamer recognition have also been used for pesticide sensing and include the most common surface-enhanced raman scattering (SERS) or resonance light scattering (RLS) and other techniques (Wang et al., 2016; Nie et al., 2018).

Compared to antibodies, the aptamer recognition method presents significant advantages for the quantitative detection of small-molecule pesticides. Properties like high specificity, affinity, and simple nucleic acid aptamer modification contribute to their comprehensive and intelligent adaptation in the field of pesticide residue detection. In addition, aptamers are equipped with stable recognition activity and they are accessible and economical, making them competitive candidates for detection product development.

## Nanomaterial-Based Recognition of Pesticide Compounds

In recent years, nanomaterials have attracted considerable research attention in fields like material science, nanomedicine, computer science, and chemical catalysis. Many nanomaterials are currently used for identification rather than merely for signal output or signal amplification.

A molecularly imprinted polymer (MIP) represents a chemical antibody that specifically binds to chemical molecules. Due to its high mechanical properties, superior stability, simple preparation on a large scale, and reutilization potential, MIP is widely used in the field of sensing (Wulff and Sarhan, 1972). It can be combined with various materials possessing output signal properties for pesticide detection. For example, in 2020, molecularly imprinted upconversion nanoparticles were constructed for identifying acetamiprid (Yu et al., 2020) with a detection range of 20 ng/ml to 800 ng/ml and a LOD of 8.3 ng/ml, confirming the excellent detection potential of the sensor (Figure 1E). Moreover, MIP can also be utilized in conjunction with carbon dots, metal nanoparticles, and fluorescent molecules (Li et al., 2015; Feng et al., 2019; Kazemifard et al., 2020).

Metal-organic frameworks (MOFs) are constantly explored due to their structural consistency, controlled synthesis, and unique chemical properties (Zhou and Kitagawa, 2014). An appropriately designed MOF skeleton can be identified based on host-guest interaction and specific molecule binding. Xu et al. (2020) developed a zinc MOF (Zn-MOF) with luminescent ligands, as shown in Figure 1F. The optical sensor enabled selective parathion recognition and super-sensitive detection with a LOD of 1.950 ppb. Moreover, other nanomaterials like metal nanoplatelets can also be used to rapidly detect pesticides (Thirumalraj et al., 2020).

Nanomaterials have received considerable attention for molecular recognition due to their excellent specific surface area and stability. Furthermore, they are simple to produce *in vitro* on a large scale at a low cost, with multiple functions regarding catalytic chemical reactions and signal output. However, as a developing field, the cross-reactivity with untargeted pesticide molecules and specific identification by nanomaterials requires further investigation to create ideal POCT products for pesticide detection.

## Macrocycle-Based Recognition of Pesticide Compounds

Macrocycle-based methods for identifying pesticides often combine macrocycles with fluorescent elements.

Non-fluorescent macrocycles reduce the distance between the pesticide and fluorophore, modifying the fluorescence energy, and producing the signal output to complete pesticide detection. Zhang et al. (2020) synthesized three porous organic polymers modified by different carbazole groups to prepare pesticide test papers. Those polymers can identify pesticides and react with fluorescent signals. Energy transfer occurs between the pesticide molecules and the carbazole group on the polymer, reducing the fluorescence intensity.

Another strategy for pesticide detection using macrocycles involves replacing fluorescent molecules with pesticides to turn on fluorescence. Xu et al. (2021) used pesticides to replace the protonated acridine (AD) in the macrocycle to detect dodine (DD). First, synthesized cucurbit [10] uril (Q [10]) containing AD in the cavity was synthesized, which inhibited the AD fluorescence intensity. In the presence of DD, it can displace AD from the cavity of Q [10] to enhance fluorescence.

## Discussion

Various specific identification elements, such as antibodies, enzymes, aptamers, and nanomaterials, can be used for POCT pesticide detection. The traditional enzyme-based pesticide identification method relies on the specific properties and buffer system of the enzyme. Sensors based on this principle must be improved in terms of stability, specificity, and selectivity. However, biosensors based on non-enzyme recognition display superior affinity, specificity, and stability with no restrictions regarding pesticide types. Furthermore, as an emerging identification approach, nanomaterials display superior stability, unique catalytic activity, and photoelectric properties, warranting attention in molecular recognition. Therefore, although pesticide biosensors developed based on different recognition modes can meet the high specificity, and sensitivity requirements for detecting pesticides in complex samples, developing more advanced identification techniques is essential.

POCT products, such as flow cytometry test strips, microfluidic devices, and detection kits can achieve the on-site detection of pesticide residues based on liquid systems or solid-phase devices. In addition to fast, sensitive, and stable detection, it is necessary to adapt the detection device to different sample matrices and simplify the sample pre-processing steps to enhance the on-site detection performance. This allows the development of sensor devices able to meet market demand.
